# Enteral Green Beans in the Management of Neonatal Irritant Diaper Dermatitis

**DOI:** 10.1111/pde.70163

**Published:** 2026-02-16

**Authors:** Allison Chiou, Lindsay Tufte, Matthew Mahoney, Christina Boull

**Affiliations:** ^1^ Medical School University of Minnesota Minneapolis Minnesota USA; ^2^ Department of Dietetics M Health Fairview Masonic Children's Hospital Minneapolis Minnesota USA; ^3^ Department of Dermatology University of Minnesota Minneapolis Minnesota USA

**Keywords:** fiber supplementation, irritant diaper dermatitis, neonatal diaper rash, neonatal nutrition, pediatric dermatology

## Abstract

Irritant diaper dermatitis (IDD) is frequently observed in neonatal intensive care units and is often exacerbated by chronic loose stools. Despite standard treatments, erosive or persistent IDD remains challenging to manage. We introduced pureed green beans into enteral feeds as a source of fiber to improve stool consistency and reduce skin breakdown. This intervention has been highly effective as an easy‐to‐use and safe non‐topical intervention for treating IDD.

To the Editors,

1

Irritant diaper dermatitis (IDD) is commonly observed in the neonatal intensive care unit (NICU) [1]. IDD classically occurs due to chemical irritants, mechanical friction, and an alkaline pH that increases fecal proteolytic enzyme activity, causing immature epidermal barrier breakdown and increased susceptibility to secondary infection [1]. NICU patients are particularly vulnerable to IDD due to early stool contact, medical interventions, and high‐concentration feeds leading to osmotic diarrhea [2].

Management of severe IDD focuses on skin barrier restoration by (1) removing skin irritants with frequently changed superabsorbent diapers or diaper‐free time, (2) gentle cleaning of the diaper area with minimal friction, and (3) topical therapies such as barrier creams, topical corticosteroids, and antimicrobials for secondary infection [3]. Despite these measures, treating severe erosive IDD remains a challenge when frequent loose stools are the primary culprit.

At our institution, enteral green bean supplementation has emerged as a supportive strategy in managing refractory IDD associated with frequent loose stools. This practice was adopted in 2012 with a protocol based on a study from Children's Hospital of Illinois, where green beans improved stool consistency and reduced parenteral nutrition dependence in infants with short bowel syndrome [4]. In our broader patient population, green beans were found to be accessible, easy to use, safe, and effective at bulking stool in neonates with frequent loose stools. We have noted improvement in many infants with IDD within a few days of starting supplementation. A representative example is shown in Figure 1.

**FIGURE 1:**
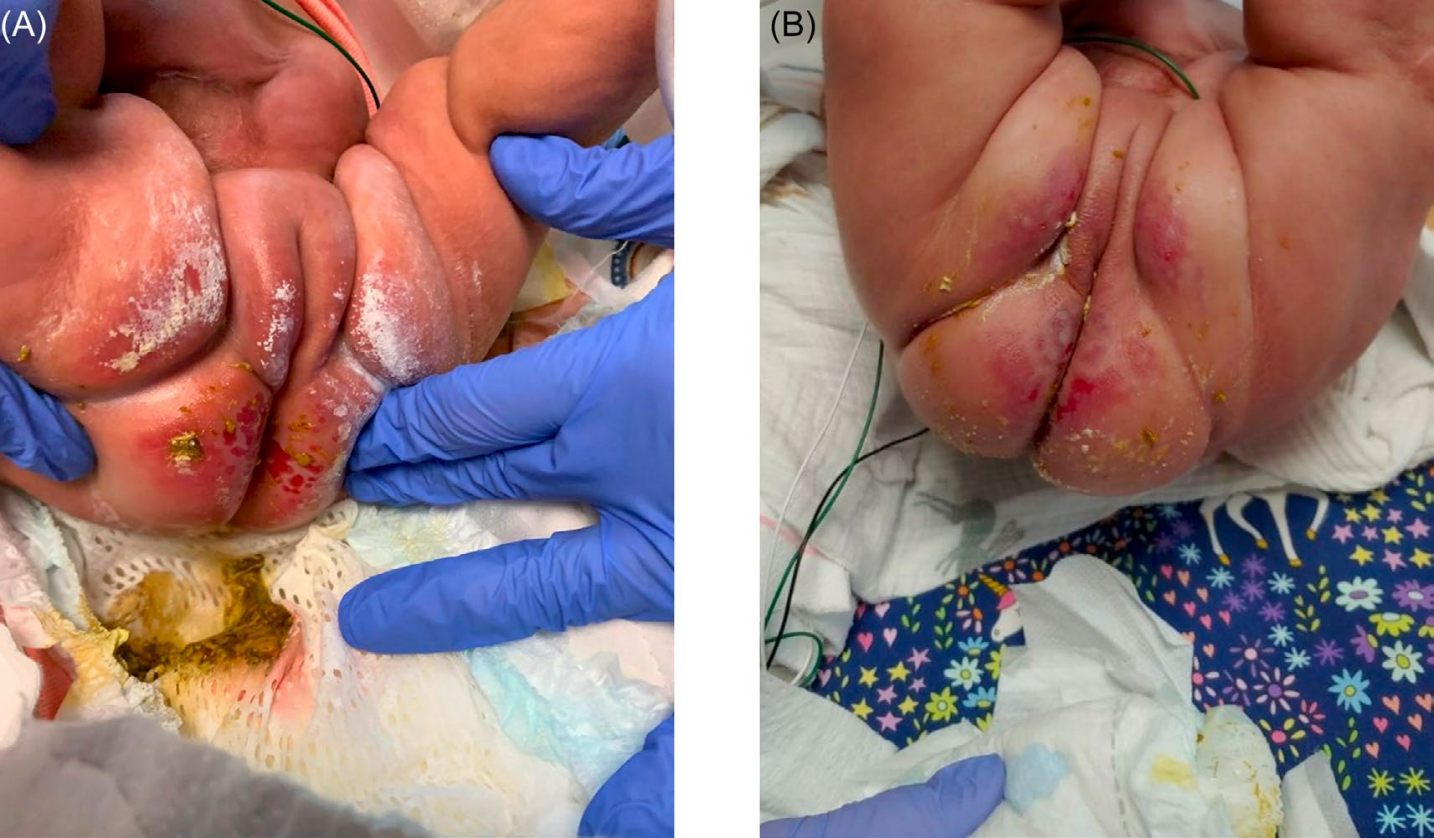
Neonate with irritant diaper dermatitis successfully treated with the addition of green bean puree to enteral feeds. Panel (A) is prior to addition of green beans and shows significant erythema and well‐circumscribed eroded papules on the bilateral buttocks and perineal area. Panel (B) demonstrates significant further improvement of irritant diaper dermatitis 13 days after the initiation of enteral green beans.

Our protocol suggests that infants be at least 37 weeks corrected gestational age (CGA), as fiber is generally avoided in premature neonates due to concerns about intestinal immaturity [5]. Neonates receive pureed green beans mixed with formula or breast milk during their regular enteral tube feeds. Initially, 30 mL (1 oz) of green bean puree (Gerber Stage 2, ~0.5 g of fiber/oz) is added to each 240 mL (8 oz) of formula or breast milk. The green bean content is then gradually increased by 30 mL (1 oz) every 2–3 days until the desired stool consistency is obtained.

There are no absolute contraindications to the use of green beans, but infants with complex gastrointestinal (GI) histories (such as necrotizing enterocolitis or recent GI surgery) are assessed carefully, often in consultation with pediatric surgery and gastroenterology. Infants with gastrostomy tubes can experience clogging, but this can typically be avoided with proper mixing. Caloric displacement must be considered, as green beans have significantly lower caloric density (~10 kcal/oz) than formula or breast milk and require feeding rate adjustments to accommodate added volume. We advise working with a NICU dietitian to implement green bean supplementation.

In summary, enteral green beans represent a low‐cost, accessible, and generally well‐tolerated adjunct for managing refractory IDD due to frequent loose stools in the NICU. While our institutional experience suggests substantial benefit, definitive proof of efficacy would require confirmation in a randomized controlled trial.

## Conflicts of Interest

The authors declare no conflicts of interest.

## Data Availability

The data that support the findings of this study are available from the corresponding author upon reasonable request.
